# The Intersectionality of Race and Trajectories of African Women into the Nursing Career in the United States

**DOI:** 10.3390/bs10040069

**Published:** 2020-03-25

**Authors:** Linda L. Semu

**Affiliations:** Department of Sociology, McDaniel College, Westminster, MD 21157, USA; lsemu@mcdaniel.edu

**Keywords:** intersectionality, globalization, migration, nursing, race, gender, discrimination, language

## Abstract

This paper uses narratives of Malawian-born registered nurses working in the United States to capture pathways through which African women are entering the nursing profession. The paper highlights how race, immigrant status and language acts as potential sources of discrimination within the nursing profession. The paper utilizes intersectionality as a feminist framework that places black women’s experiences at the center of analysis to capture the multidimensionality of their experiences. The qualitative study highlights the multiple pathways through which African immigrant women enter the nursing profession and how being African, immigrant female nurses predisposes them to discrimination in their interactions with employment institutions and patients. Focusing on African women’s experiences as recent immigrants enriches the global migration narrative and helps contextualize the intersectionality of race, gender and discrimination within particular contexts.

## 1. Introduction

This article is part of the growing literature that is focusing on immigrant African women’s experiences as part of the global migration narrative which brings to focus their identity as black, immigrant and female in the workplace [1,2]. Research on migrant nurses’ experiences of racism and marginalization in the workplace [2,3,4,5,6,7] has mostly focused on Asian health care workers. Research specific to black African female nurses’ experiences has mostly been conducted in Canada [8,9] and the UK [10]. Recent work by Showers [1] shows that for West African immigrant women working in US health-care settings, their racial and ethnic identities are potential sources of discrimination. This article contributes to this genre of research. The findings analyzed in this article are based on a qualitative study that utilized snowball sampling to solicit responses from Malawian-born registered nurses working in the United States on a questionnaire with closed and open-ended questions, followed by discussions and in-depth interviews. Increasingly, qualitative data collection is being utilized to capture the nuances of the immigrant experience and narrative. For example, Showers conducted in-depth interviews with forty-two female registered nurses living and working in Washington DC from the top four sending countries of immigration in West Africa: Nigeria, Ghana, Sierra Leone and Liberia [1]. In the United Kingdom study of registered nurses in the National Health Service, 30 semi-structured interviews and focus group discussions were conducted among Black African nurses from Malawi, Kenya, Ghana, Nigeria, South Africa, Zambia and Zimbabwe who were recruited through advertisements [10]. Vukic, Jesty et al.’s 2012 study to illuminate the interplay of race and racism among 22 indigenous nurses in Canada was based on snowball sampling and qualitative interviews [11]. Dicicco-Bloom’s 2004 study relied on snowball sampling to recruit 10 South Asian nurses from whom data was collected through semi-structured interviews and open- ended questions [7]. This article is based on analysis of qualitative data of 11 Malawian-born female Registered Nurses (RNs) who were part of a larger study of Malawian-born health-care workers in the United States. The larger study sought to highlight immigrants’ professional and cultural adaptation as they negotiate their national/ethnic/racial identity in the US healthcare system. This article explores the pathways through which they enter the nursing profession and how they navigate experiences of discrimination through the intersectionality of being African, immigrant and female in their interactions with employment institutions and patients within the US health care system. There is a paucity of research on African immigrants in the United States. Africans are a relatively small immigrant group and have had a short history of voluntary migration to the US. African women have been studied even less. That notwithstanding, Arthur [12], shows that African women are active and independent players responding to the same geopolitical, economic and social forces at the core of the movement and transfer of human capital and labor from the developing to the developed world. Arthur argues that West African women use international migration as a strategy to achieve economic empowerment, challenge traditional norms and rigid gendered structures, attain self-defining autonomy and forge new identities and alliances across geographical centers. More research is definitely needed, and first-hand reports of lived experiences must be added in order to come up with a holistic understanding of the immigration narrative of Africans in general, and African female nurses in particular [2]. Two main theoretical strands help contextualize the narratives: the intersection of gender, race and immigration in the era of globalization and the issue of immigrant care work as it relates to the nursing profession.

### 1.1. The Intersection of Gender, Race and Immigration in the Era of Globalization

Globalization and the subsequent increase in movement has intensified strangeness while simultaneously normalizing it as ideas of race and racism are either confirmed or redefined within the context of inequalities [13,14]. International airports have become spaces for asserting national identity, reproducing racism and reinforcing the differential status of people in the global economy. As such, most Black Africans traveling to Europe and North America have increasingly been subjected to extra scrutiny, surveillance and searches [15]. Race has become a salient factor through globalization and migratory flows from former colonial subjects in the global South to the global North, whereby racialized ethnicity is used to regulate movement [16]. Globally, there are 272 million migrants, representing 3.5% of the total global population. Furthermore, 74% of the international migrants are of working age (20 to 64 years of age), of whom 48% are female. Most international migrants are from the global South to the global North [17]. It is therefore imperative that transnational migration studies must utilize analytical frameworks that examine the interconnection of different identities and hierarchical structures relating to gender, ethnicity, race, class and other social divisions at national, transnational and global levels. As an analytical framework, an intersectional approach emphasizes how different forms of discrimination co-constitute each other to produce particular outcomes of vulnerability and inequality [18].

Intersectionality is informed by the view that various dimensions of social life cannot be separated into discrete strands but rather that people are socially located along multiple axes such as gender, race, class, ethnicity, stage in life cycle [19,20]. This multiplex analytical frame has a long history in the US [21,22,23,24]. An intersectional analytical framework incorporates the way in which race and gender interact to shape the multiple dimensions of Black women’s employment experience [24]. It is a methodology that utilizes thought and action to generate theory by allowing concrete experiences to challenge the worldview offered by theory [23]. According to Collins, intersectionality comprises three inter-related dimensions: (i) a field of study (object of investigation); (ii) an analytical strategy for investigating social phenomena; and, (iii) a critical praxis for connecting scholarship with social justice [22]. Showers’ analysis of the literature on the intersections of gender, race, class and nation in the study of migration highlights four key tenets that the scholarship highlights: (i) the terms of incorporation in receiving societies; (ii) how black immigrants fare in the labor force; (iii) although black African immigrants, on average, possess high levels of education and human capital, their earnings fall below what their educational credentials might suggest; (iv) it is important to understand gender as a core organizing principle of migration [1]. This paper fleshes out these intersectionalities by highlighting the educational pathways into the nursing career, and analyzing experiences of structural and individual discrimination of Malawian immigrant nurses in the United States.

### 1.2. Immigrant Female Care Work: Trends in the Nursing Profession

The ‘global care chain’ concept describes the experiences of female immigrant domestic workers by showing that people across the globe are linked through care work by people of color from poor countries in rich countries [25]. This concept is also applicable to the nursing profession. This is because race, gender and ethnicity shape the labor market experiences of African immigrant women as well as the overall diversity experiences of black immigrants in the United States [1], more so since nurses have become part of the global migratory stream [26]. With 51 million foreign-born nationals, the United States is host to the largest migrant stock of any country worldwide [17]. Immigrants constitute 16% of healthcare labor force in the United States. The most common healthcare jobs for naturalized citizens include: registered nurse (19.8%); nursing/ psychiatric/ home health aide (18.4%); and physician/ surgeon (11.4%). The most common healthcare jobs for noncitizens include allied health occupations such as: nursing/ psychiatric/ home health aide (27.3%); personal/ home care aide (17.6%); registered nurse (12.7%) [27]. While the gradation highlights the increased likelihood of social and labor market vulnerability of non-citizens compared to naturalized or native-born citizens [27], it serves to direct us to the fact that the global economy has made it possible for gender ideologies to support and naturalize arbitrary claims about what kinds of bodies are best suited to particular tasks [28]. As more women become migrants in their own right, they are among the most highly skilled migrants, especially as nurses have become a critical resource in an increasingly competitive global labor market. A perfect storm of nurse migration has been created by the simultaneous occurrence of events in developed and developing countries. Nurse shortages in industrialized countries driven by an ageing population, relative job security and higher wages are generating demand. Simultaneously, conditions in many developing countries have pushed thousands of nurses to migrate in search of better pay and working conditions, career mobility, professional development and a better quality of life [19]. The nurse shortage in the US is projected to reach 1 million by the year 2022, fueled by demand, retirement, others leaving the profession and nursing schools that are ill-equipped to train adequate number of nurses [29].

The current United States registered nursing stock of 3,059,800 is inadequate to meet demand. The Bureau of Labor Statistics projects that for the 2018–2028 period, employment of registered nurses will grow by 12%, making nursing the top occupation for job growth [30]. With a median pay of $71,730 [30], the profession has become a viable option for internationally educated nurses—IENs [31]. The Philippines is the major source country of the IEN workforce (50%), followed by Canada (12%), India (9.6%), the United Kingdom (6%), Korea (2.6%), Nigeria (2.1%) and other (17.8%) [32]. Nigeria is the only Sub-Saharan African country among the top 10 source countries for the US [33]. That notwithstanding, many African countries have a significant proportion of their nurse stock working in industrialized countries. A total of 65,000 professional nurses from Sub-Saharan Africa are working in nine key destination countries: Canada, France, Portugal, South Africa, Spain, Belgium, Australia, the United Kingdom and the United States. Liberia, Burundi and Mauritius have the highest proportion of their nurse stock working abroad, at 81%, 78% and 63%, respectively, while Egypt (1%), Sudan (1%) and Niger (2%) have the lowest proportion of nursing stock working abroad. A total of 17% of Malawi’s 2248 nurses are working abroad, mostly in the United Kingdom and the United States (171 each), followed by: Australia (14), South Africa (11), and Canada (10) [34,35].

Registered Nurses (RNs) in the US are predominantly non-Hispanic White. Although the number of minority racial/ethnic RNs increased from 333,368 in the year 2000 to 513,860 in 2008, the racial/ethnic distribution of the RN population is substantially different from the general population [32]. The 2017 National Nursing Workforce Survey found that only 19.2% of RNs are minorities, including those with two or more races distributed as follows: Asian (7.5%), Black/African-American (6.2%), other (2.9%) and two or more races (1.7%). The proportion of Black/African-American RNs falls below the 13.3% black national population, while the Asian proportion of nurses is higher than the national population proportion (5.7%) [36]. Foreign-born immigrant nurses are increasing the diversity of this sub-sector of the labor force. The shift in complexion and their accents has made them visible, more so since hiring foreign nurses is no longer a short-term solution, but instead is now a long-term solution to the nursing shortage problem in the US and other industrialized countries [37]. In the context of increasing demographic diversity, information about the experiences of individuals from various groups is necessary so as to illuminate the intersection of immigration, gender, race and language [7]. Studies of immigrant nurses have found language and communication; marginalization, discrimination and exploitation; as well as racism, cultural dissonance and resilience as emerging themes that need to be further explored in order to understand the lived experiences of these nurses [2,5,6,7,38]. Therefore, this article, which is based on Malawian-born RNs working in the US, highlights: the multiple ways in which the women enter the nursing profession, often having to adapt their educational and professional choices, irrespective of prior training or intended educational goals at the time they migrated to the US; the appropriation of a niche as a strategy to overcome entry barriers to the US labor market; the intersection of the nurses’ nativity, race, gender and language and how these mediate their experiences of discrimination structurally and interpersonally with their American colleagues and patients.

## 2. Methods

This study aimed to produce narratives of the immigration and subsequent professional experiences of Malawian healthcare workers practicing in the United States. Authorization was obtained from the Institutional Review Board to ensure all protocols for the protection of research participants were set in place. Participants in the study were all adults (above 18 years old). They were all informed of confidentially and other protections, as well as informed consent that was obtained before the onset of interview, irrespective of whether it was the initial or subsequent follow-up interviews. The study was conducted in two phases: A baseline study of Malawian-born healthcare workers in phase 1, followed by an in-depth study of Malawian born registered nurses in phase 2. Participants in phase 1 of the study were from a broad range of medical professions: medical doctors, psychiatrists, pharmacists, dentists, nurses and laboratory assistants. Snowball sampling was utilized to recruit participants. An initial contact was made via email announcements to a list-serve of US-based Malawians, followed by contacts via phone, email and references from respondents who knew of others in the healthcare industry. Thirty Malawian immigrant health-care workers from across the US were contacted as potential research participants of whom 18 participated in the study. Phase 1 comprised a baseline survey that contained both closed and open-ended questions on: demographics (age, marital status, number of children and education); professional profile, working conditions and experiences of discrimination. The professional profile section sought information on: qualifications; reasons for working in the United States; training in the healthcare industry prior to migrating to the United States; and where applicable, why they shifted to health care; length in current position and service location. Questions on working conditions, experiences, and transcultural interactions sought to obtain information on the respondents’ perceptions related to: (a) their job (whether they felt their skills were utilized, shifts, pay, subjection to scrutiny, job security) and (b) their experiences interacting with patients and colleagues. Organizing the research instrument thematically as a strategy to capture experiences of immigrant nurses has been utilized by researchers, including Xu [5,6] whose themes covered: communication; differences in nursing practice; marginalization, discrimination and exploitation.

Initial review of descriptive data showed that the majority (11) of the participants (18) were female registered nurses (RNs). The RNs were then engaged in phase 2 of the study that comprised: a detailed analysis of their responses from phase 1, follow-up discussions and interviews to gather more in-depth narratives of their life and career trajectory. This article is based on analyses of data from the 11 RNs. This triangulation of data collection (survey and in-depth interviews) is similar to Showers’ strategy where she utilized in-depth interviews and field notes from participant observation to study West African immigrant women nurses working in the United States [1]. In-depth interviews of Malawian-born nurses lasting between 30 and 60 minutes each enabled the participants to narrate their educational and professional biographies and other pertinent issues. Interview notes were analyzed and observations recorded immediately after each interview. Commonalities in responses showed that the study had reached a certain level of saturation for which further data collection became redundant. Data analyses were informed by the inductive paradigm and grounded theory. The inductive paradigm is based on a person’s view of reality (meanings), with which actions are defined and drawn from events and experiences [39,40]. This enables researchers to approach the data with an open mindset as they discover what it reveals [41]. Globalization and its attendant cultural diversity have resulted in the widespread use of grounded theory since the approach enables researchers to: understand what is going on; account for participants’ main concerns; and to see underlying uniformity in otherwise disparate facts [42]. The main concern in the analysis was to capture depth and consistency in the data. This involved looking at the descriptive statistics so as to identify emerging patterns. Data from open-ended questions and interviews were analyzed through a process of open coding and constant comparison, followed by axial coding in which relationships among concepts and categories were identified [41]. In addition, detailed research memos were written to facilitate analysis. Findings of this study are corroborated by studies that have been conducted on immigrant nurses in the US, Canada and the UK. The findings clarify the pathways through which Malawian women find themselves as RNs and their experiences of discrimination as they confront the intersection of gender, race, and language within their employment institutions and with their colleagues and patients. Participants (RNs) were given a copy of the report of the findings for comments. They expressed appreciation for how their personal experiences were respectfully and skillfully woven into a broader narrative capturing the intersection of gender, race and language in the face of globalization.

## 3. Results

### 3.1. Sample Description

Qualitative analyses and descriptive data (Table 1) are based on the Malawian-born RNs practicing in the United States. They range in age from 31 to 50, half of whom are aged 40 and above. This is similar to US national data where the median age for RNs in 2018 was 47.9, while 53% of the nurses were aged 50 and under [43]. Most of the RNs in the study are married with children, have stayed in the US for over 10 years and they all work within urban/ metropolitan areas and surrounding suburbs. One of the respondents had worked at the same position for more than 10 years; 5 had worked in their current position for 1–3 years; and only 2 had worked as nurses for more than 15 years. Most of them acquired their nursing qualifications after the year 2000. It will become apparent in subsequent discussion why they have worked as nurses for shorter periods despite being in the US for much longer.

Schumacher’s 2011 analysis showed that foreign-born nurses that are new to the US have 4.5% lower wages compared to native and Canadian born nurses, although this wage disadvantage disappears after 6 years [44]. However, Lowell and Gerova [4] argue that compared to native-born RNs, international RNs generally earn 18% more, are more likely to have a Bachelor’s degree and they tend to work slightly more hours per week on average. This study corroborates the latter finding, especially when compared to results from the National Sample Survey of Registered Nurses in the United States. Among the native-born RNs, 45% have an Associate Degree in Nursing (ADN); 34.2% have Bachelor’s or graduate degrees while 20.4% received their initial education in hospital-based diploma programs. Overall, 63.9% are college educated and 19.3% have graduate degrees (masters or doctorate) [31,43]. All the Malawian-born RNs are college-educated, 45% of whom have an ADN while 55% have a Bachelor’s degree or higher. According to the 2018 survey, median earnings in the US for full-time RNs were $73,929. The highest earnings were as follows: Nurse Anesthetists (NAs) averaged $161,076; Certified Nurse Midwives earned $102,115; while Nurse Practitioners averaged $99,962. On average, male nurses earned more ($79,928) than female nurses ($71,960) [43]. This is similar to the trend shown by Malawian-born RNs (Table 1) where the top three earners have graduate degrees and are at the administrative/management level. The earnings for these nurses are a far cry from the annual earnings they would hope to make if they worked in Malawi: $2,636 for ADN and $4510 for RNs. It is not surprising therefore that although nursing is a gender segregated job, it has become the career of choice for many immigrant women in the US.

The pay notwithstanding, results from the study show that as Black female African immigrants, these Malawian-born RNs are vulnerable to discrimination at multiple levels in the course of performing their duties. This paper examines these experiences through the two thematic trajectories of: (a) education and pathways into nursing; and, (b) experiences of discrimination through intersections of being non-white immigrant female nurses in their interactions with employment institutions, their colleagues and patients. These two phenomena highlight the key analysis in intersectionality: the interviewees’ gender, race/ethnicity and class on the one hand, and power relations within the context of institutionalized racism/ discrimination on the other [20].

### 3.2. Education and Pathways into Nursing

Migration is a multi-faceted process with plural routes that involves nonlinear and multidirectional movement [45]. All but 3 of the 11 RNs had no prior training in nursing before immigrating to the US. Among those three, one applied for a nursing job while in Malawi and the other two obtained their jobs while in the US where they were pursuing further training. They decided to apply for a job: to gain experience and for better pay and working conditions. However, it is in the narratives of the US-trained RNs that various pathways to a nursing career emerge. Some relocated to the US for graduate education while for others, it was to acquire their first post high-school training. Upon arrival in the US, they acquired training in their desired fields: accounting, agriculture, business administration/ management, computer programming, education and natural sciences. However, they experienced typical deskilling that occurs in the process of migration: many professionally trained and experienced racialized immigrants are vulnerable to socio-economic constraints that are characterized by low-end, precarious forms of employment that marginalize them in terms of income and status [8]. They could not find jobs/ employers willing to give them a chance in their chosen fields therefore they had no choice but to switch to nursing post-graduation. As a result, two-thirds of the RNs have 17+ years of schooling. Despite their high qualifications, some of the women worked at some point as Certified Nurse Assistant (CNA) and Licensed Nurse Practitioner (LPN), the lowest ranks in the nursing hierarchy. This highlights the class axis in intersectionality: most African immigrants (including women) are usually from middle class backgrounds in their home countries, yet they experience racism and downward class (social and occupational) mobility when they immigrate to the US [1]. A 2014 analysis of Asian students’ education-migration experiences in Australia shows that the uncertainty and precariousness inherent in the student-to-migrant process creates significant tensions in the daily lives of most student-migrants, both as individuals and as members of transnational families with long-term collective migration strategies [46]. This is corroborated in this study and the following vignettes capture the twists and turns of immigrant Malawian women to the United States in their pursuit of an education and their path toward a nursing career. All names and personal identifiers have been changed to protect participants’ identity.

#### 3.2.1. Dalia and Tia: Twists and Turns on Their Way to Becoming RNs

Dalia and Tia are RNs each with an associate degree in nursing (ADN), obtained in 2011 and 2008, respectively. Dalia is 47 years old and is very passionate about teaching children. She acquired primary school teaching certification in Malawi in the mid 1980s, where she taught for several years and operated her own private school. In 2001, she and her children relocated to the US, where she attained an associate degree in early childhood education. She had acquired certification as a CNA while she was a studying for her education credentials as a means to support herself and her children. However, since she could not get a job in her field despite her college credentials, she decided to take up nursing as a career. She acquired certification as an LPN and got a job commensurate with that training. She then enrolled in an ADN program from which she graduated in 2011. It took her 10 years from her arrival in the United States to finally attain a financially stable career. Although the road took some unexpected turns, she is happy with the choice she made. She is hoping to pursue a BSN at some point in the near future.

Although the end result is the same, Tia’s story is somewhat different from Dalia’s. Tia is 41 years old. She came to the United States in the early 1990s to study culinary arts right after graduating from high school in Malawi. She spent the first 6 years trying out various programs, after she decided not to pursue culinary arts. At times, she would go for a semester or two without enrolling in school due to financial constraints, as her family could no longer afford to financially support her from Malawi. She eventually acquired CNA certification, which enabled her to support herself and her two young children. The work was backbreaking but better than the minimum wage jobs she had held prior to the certification. She then enrolled in a part-time LPN program. After acquiring her certification, she worked to clear her debt and then enrolled in the ADN program from which she graduated 5 years later. It took her 17 years from her arrival in the United States to becoming an RN, which has enabled her to have a sense of professional accomplishment, economic stability and empowerment. This circuitous pattern has also been observed by Robertson’s studies on Asian migrants to Australia, where he concludes that the experiences of migrants increasingly involve complex trajectories that encompass multiple transitions across statuses and places to produce specific experiences that challenge normative life transitions and migration outcomes [47]. Clearly, each migrant narrative demonstrates that patterns of migration are heterogenous, as seen by Lala and Nia’s experiences, who are both seeking to acquire advanced degrees.

#### 3.2.2. Lala and Nia: Looking to Get Advanced Degrees beyond BSN

Lala and Nia are both RNs with BSN degrees obtained in 2008 and 2007, respectively. Lala is 38 years old. She obtained a Bachelor of Science degree (Biology) from the University of Malawi in the mid 1990s. She taught secondary school biology and math for two years, then she relocated to South Africa where she taught math for two years. She then relocated to the US in 1998 where she acquired a Bachelor’s degree in computer programming. However, she was unable to get a job. She returned to Malawi in 2002. In 2004, she re-entered the US and enrolled in a BSN program from which she graduated Magna Cum Laude in 2008. She later obtained a Master’s degree in clinical leadership and nursing education. She is currently working at a teaching hospital and is also an adjunct lecturer in nursing. Her goal is to obtain a PhD in Public Health, which she hopes will enable her attain a full-time academic position.

Nia’s relocation to the US was not as circuitous as Lala’s. At 32 years old, Nia is one of the two youngest women in the study. She relocated to the US in the late 90s, soon after graduating from high school. She obtained a bachelor’s degree in agriculture. Nia has an older sister who has worked as a nurse in the US for more than 20 years. Immediately after graduation, when she could not get a job in her field, she enrolled in a BSN program, from which she graduated in 2007. Thereafter, she acquired a job as an oncology nurse. She went on to obtain Chemotherapy and Biotherapy Certification with the National Institutes for Health (NIH). She is currently enrolled in a Master’s Degree program.

According to the National Sample Survey of Registered Nurses, more than 21% of RNs earned an academic degree prior to their initial nursing degree [31]. This is particularly true for foreign-born women who obtain their initial nursing training in the US. The narratives have shown that these women’s journeys are not as straightforward as one would expect. Although their academic and career aspirations were far removed from the nursing profession when they first arrived in the US, they underwent what Mensah [14] refers to as “job/status identity crisis” that exposed them to economic exclusion. Instead of pursuing the fields in which they were initially trained, most immigrant Africans engage in “survival employment” [48]. The Malawian RNs have now become part of the subset of migrant women workers who engage in various aspects of nurturing and maintaining people’s well-being [36]. The training takes a long time because they are self-sponsored: most enter the United States as students hence, are not eligible for federal and other funding. Consequently, they start from the bottom of the nursing educational and career trajectory: from CNA to LPN, then ADN, and for some BSN and beyond. In our follow-up interview, Lala elaborated on this process by narrating the experience of her classmate: A Ghanaian woman in her late 50s whose journey has taken 12 years as she subsequently moved up from CNA to LPN to RN and is now about to get a graduate degree.

These women have responded to an increasingly gendered and ethnically segmented labor market where the nursing profession has become the new ghetto for immigrant women of color. Ironically, as they provide this transnational labor, they themselves are pursuing globally inflected desires for class mobility and consumption [27]. All the women have full-time jobs which they obtained during their nursing training or immediately after graduation. These women are not only taking care of themselves and their children, but have also become part of the global remittances chain whose value has become more than double the size of international aid flows in recent years, accounting for considerably more than foreign direct investment in Africa [25]. Although previous research focused on experiences of IENs working in the US, the narratives of these women show that foreign-born nurses trained and working in the US have just as, if not more, compelling stories of strength and resilience which should be told. These narratives demonstrate that although education offers migration pathways to international students, the student-migrants face periods of extended precarity as they deal with complex forms of inclusion and exclusion from citizenship and shifting goal posts in terms of long-term aims for permanency. However, the narratives express agency and resistance in ways that may be trans-formational even as they reaffirm the same neo-liberal discourses about citizenship and migration behind the policies that structure the nurses’ experiences [49].

### 3.3. Language and Competence as Codes for Discrimination Against Visible Minority Immigrant Nurses 

Gender, ethnicity and immigrant status intersects to shape visible minority women’s employment experiences. This is more so for foreign born minority women, as they are likely to experience more difficulty in the labor market due to their immigrant status and cultural barriers that can impede their economic progress [50]. Black African immigrant nurses experience discrimination in institutional health-care settings at interpersonal and structural levels. Discrimination, the process of treating someone less favorably than another person because of an identity group characteristic—negatively impacts individuals and organizations, especially where multiple forms of marginalization intersect and operate simultaneously [51], as is the case for the immigrant Malawian women RNs in this paper. Structurally, nurses who are African immigrants and other women of color are concentrated in the least desirable specializations within hospitals and units. This limits their opportunity in the workplace and acts as an obstacle to upward mobility [1,10]. At the interpersonal level, discrimination is experienced from their interactions with patients and patients’ families where questions of belonging, identity and difference are forms of boundary and hierarchy-making that are central to inequalities [52]. By virtue of their nativity, race and accent, international nurses may encounter: (i) marginalization and lack of support from peers, supervisors and managers; (ii) unfair treatment and racism in the form of stereotyping and rejection by patients [5,9]. RNs in the study experienced these forms of discrimination, hence their foreign status is salient in the ways they identify as immigrants to the United States [1].

#### 3.3.1. Structural Discrimination

Structural racism, the totality of ways in which society fosters racial discrimination through mutually reinforcing beliefs, values, practices and patterns [53] is the norm rather than the exception in the experiences of Black African immigrant nurses [1,9,10]. In the current study, respondents reported the tendency for male nurses to be favored and given management positions faster than female nurses, although the profession is female dominated. Although this is applicable to all female nurses irrespective of race and nativity, African immigrant nurses’ gender intersects with their race, nativity and speech patterns to affect their experiences in the workplace, especially in matters of equal opportunity. Patterns from the data show there are five ways in which structural racism is manifested (both covertly and overtly): deskilling, type of assignments, excessive monitoring, pay inequity and linguistic barriers. Two thirds of the respondents are familiar with US labor laws, especially those related to overtime compensation, equal employment opportunity and pay. However, there is a perceived gap between the legal provisions and their experiences as seen in the fact they report deskilling upon taking their positions whereby their managers did not acknowledge their prior training and experiences. Lily, 45 noted that “my employer could not consider my prior training and work experiences because the companies that I worked for in Malawi are not recognized in the United States.” This in line with the inherent dissonance created by globalization’s tendency to combine and polarize skilling with deskilling [48,54]. For immigration purposes, the United States prefers people with tertiary education and yet upon entry into the US, they essentially become deskilled and have to repeat the skill acquisition process since their prior qualifications are often not recognized.

Once on the job, they report being asked to perform tasks commensurate with their skills. In addition, they become familiar with US labor laws although this does not protect them from discrimination in the form of tasks they are assigned to. Hence Drita (31 years old), a recent graduate felt that she often had to do the least desirable assignments in order to keep her job. She stated that: one gets the worst assignments that others refuse to do. Fearful of losing a job, one tends to do a lot than others.” This finding is corroborated by Showers whose research shows that African immigrant female nurses in the US are more likely to be in less desirable specialties [1].

Structural discrimination is also manifested through excessive monitoring and feelings of pay disparities. Although the RNs feel that their colleagues do not question their authority and qualifications, they still report the existence of double standards as elaborated by Nana, 45, who stated: “I feel that I am closely monitored as my supervisors feel that I’m not competent enough to get a management position mainly because I’m black.” Excessive monitoring is one of the ways Black nurses are targeted so that they feel constantly watched, judged and threatened with the prospect of being framed for dismissal [55,56]. Allan, Cowie and Smith refer to this phenomenon as “racist bullying” in which overseas nurses working in the United Kingdom experience discrimination in the form of bullying that is aggravated by racism [57]. Their research found that this form of institutional discrimination is manifested in abusive power relationships in which the manager displays poor communication and criticism towards nurses without providing constructive help to improve areas of weakness. Very often, organizations disguise the bullying by assigning the blame on the attitude of the victim rather than the manager [57]. These discriminatory behaviors escalate from microaggressions (petty harassment) to outright macroaggressions (outright conflict and hostile maneuvers from superiors) that thwart the nurses’ upward mobility [56]. Hence, the wage gap is another way through which discrimination is expressed. Although many Black nurses report that they are paid at the same rate as their native-born colleagues, some expressed doubt, citing race and nativity as important differential factors. Tama, 50, indicated that: “the typical face of an RN is a white female and I happen not to be that hence they (white colleagues) get positions in management and a higher pay.” Cheu, 46, narrated an incident where she had assumed that her compensation was at the same rate as her native-born counterparts until one day when she and a white colleague were both interviewing for a position at another establishment. They started talking and she gathered that her colleague was getting a higher rate despite their similar qualifications and experience. This is corroborated by Lopez [58] who found that highly skilled immigrant women experience an earnings penalty due to their nativity status. The high levels of incomes presented in Table 1 are therefore to be explained by the fact that foreign-born RNs tend to work longer hours than their native-born counterparts [4].

Another major marker for these RNs’ “otherness” is their accent. Although two-thirds of the respondents stated that their accent did not matter, for those to whom accent is an issue, it can be a barrier to feeling integrated at the workplace. Nita, 36, indicated that: “I feel that I am not able to articulate myself well enough because of my accent.” The problem is not mastery of the English language, but rather their different speech pattern (accent). Hence, “the nurses believed that their foreign African accents and their locations as women from Africa put them in a position of disadvantage in occupational settings and hierarchies” [1]. The communication challenge for international nurses causes a plethora of issues at work that contribute to: (i) a general sense of social rejection; (b) prejudiced responses to African accents that are used as a means of consolidating an “outsider” immigrant status that precludes a sense of belonging (social exclusion) and, can be used a tool to put others down [5,59]. Furthermore, linguistic speech patterns are often used to substitute for racial or national discrimination. Since the latter two forms of discrimination are illegal, linguistic discrimination is used as a common-sense excuse, although it is not lost upon many that most non-standard speakers of English usually happen to be minorities and/or transnational [60].

#### 3.3.2. Interpersonal Discrimination

Interpersonal discrimination is more ambiguous in subtlety than overt forms of discrimination, and yet it is not benign, and can have insidious consequences at both the individual and organizational level [61]. A study on the impact of interpersonal discrimination and stress on health among early career STEM academicians found that interpersonal discrimination leads to stress which results in decrements in performance [62]. Findings of the study showed that the experience of interpersonal discrimination has negative implications on physical and psychological health and well-being, and has larger consequences on the lingering inequalities experienced by minorities in STEM [62]. The same can be said about the immigrant RNs in this study, as they experience interpersonal discrimination in the process of their interaction with patients and patients’ families. Although at face value the incidents seem to be about accent and questioning of the RNs’ qualifications and authority, they are powerful forms of ‘othering’ and discrimination against the nurses. Apart from their visible racial marker as black women, being from Africa with accented English creates an undeniable marker of their foreign status which is compounded by stereotypical constructions of Africa as an inferior region [1]. Hence, the intersection of language, gender and migrant status creates social exclusion and discrimination. The RNs feel that patients’ questions about where they obtained their qualifications are often a subtle way of expressing assumptions that the RN does not understand the US healthcare system. They report of always having to prove themselves in order to assuage patients’ concerns. They therefore see questions about qualifications as a veiled form of prejudice.

Nana, 37, reported that “Very often, the patients and their families ask me to call for a nurse even when they can clearly see that I have an ID on me indicating my position. They don’t expect me to be in a position of authority because I’m black; especially those from the old school.” Nana went on to say: “some white folks will refuse treatment from a black person, especially a foreigner.” She also noted that “the convenient excuse some patients use is to simply say that ‘I don’t understand you’, yet I believe that they choose what they want to hear.”

Some patients hide their prejudiced views behind concerns about competency, as observed by Lulu, 49, who remarked that “some patients think we are not competent just because we have an accent. I am the charge nurse on my unit but most of the time when I go to patients they ask: you are the in-charge? How long have you been a nurse?” These experiences have also been documented among Aboriginal nurses in Canada, who reported encountering patients who preferred a White nurse because they assumed the White nurse had more training [38]. Perceptions of English fluency often translate into perceptions of competence in which accents mark the RNs as: African, immigrants, women, and less competent than those with local accents [59]. Beliefs and behaviors regarding difference, competence and discrimination intersect in complex ways such that competence is often used as a substitute terminology to hide issues that are rooted in discomfort with difference, racism and discrimination [63]. The nurses did not identify any form of institutional support to help them deal with the individual discrimination arising from interactions with patients. Quite often, management tends to deny incidents of alleged racism as being racist and rather lumps such incidents together with patients’ other negative behaviors [55]. This exonerates the institutions from having to specifically address racial issues. Similarly, a Canadian study of nurses of Caucasian descent, found that while the nurses readily identified individual incidents of discrimination and racism, they tended to distance themselves and their institutions from these incidents, and often demonstrated ambivalence when discussing discrimination at the organizational level [63]. It is such distancing, ambivalence and excusing that undermines diversity at the workplace. This denial of racism in nursing is symptomatic of the contradictions between caring, as a principle part of the identity of nursing and the embedded institutional and interpersonal racism that makes it difficult for the nursing profession to acknowledge racial prejudice and discrimination [64].

## 4. Discussion and Conclusions

African women’s experiences should be told as part of the global migration narrative [2], which is comprised of assorted experiences rather than a linear pattern of migration, assimilation and career development. This study did not set out to generate data but instead to provide narratives on educational and employment experiences of visible minority African women as immigrants in the United States. Although quantitative studies on employment experiences of minority immigrants provide useful information, more qualitative and mixed-methods studies are essential in understanding differential employment outcomes across various racial/ethnic groups [50]. This is more so since large-scale studies have tended to homogenize experiences of members of minority groups by pooling them together into one category. It is therefore essential that voices of various subgroups of visible minority immigrant populations, such as those from the Middle East, Africa, etc., are heard [50]. Findings from this study corroborate this by suggesting that quantitative data about RNs, their academic attainment and income status might not adequately elucidate the multifaceted and nonlinear professional biographies of these immigrant women. Their strategy of shifting into nursing where studying and work are simultaneously pursued re-affirms the view that the era of a single transition from full-time school to full-time work is giving way to multiple pathways, as individuals respond to globalization [54]. The women demonstrate resilience and agency by switching to nursing and staying on the jobs despite the documented challenges where on the one hand, nursing provides them a pathway to economic stability and yet is also a terrain where gender, race, immigration and language intersect to create various points of marginalization.

The women in this study all came from an English-speaking and class-privileged background, which enabled them to immigrate to the United States to begin with, and yet they experienced deskilling and were unable to find jobs commensurate with qualifications in their preferred career paths. Although nursing was not their original career choice, they feel that it is a rewarding job and they report feeling good for saving lives. They want to continue practicing in the US because wages are high and they have opportunities to advance their education [2,6]. They adapt to their work environments by concentrating on building and bringing to light their personal strengths [2]. Just like Aboriginal women in Canada, they respond to racism and discrimination by educating others [38]. When opportunities arise, they show their colleagues that they have a drive to succeed by narrating to them the struggles they have undergone in order to reach the RN stage, prompting Lala, 38, to remark that: “they see you as a role model and they are motivated to advance in their careers.” The nurses defuse the issue of accent by assuring the patients that they (the nurses) are on their side, as noted by Lulu, 49, who said: “I tell them that they should feel free to ask a question if they don’t understand what I’m saying to them because my goal is to help them understand their treatment plan and make sure they are cared for.” In other cases, some nurses just ignore blatant cases of prejudice directed at them, and instead chose to prove to their patients that they were just as capable if not better than some of their native-born counterparts. By taking on the burden of addressing the discrimination on their own, these women’s narratives are an indictment on the nursing profession and the larger societal racism where the axes of being a woman, an immigrant, non-white and non-native English speaker intersect to create marginalization [7]. In principle, nursing’s attribute of empathy should be a hindrance to racism. However, in practice, empathy is operationalized at the individual level as an orientation to providing care. In essence, this absolves institutions from having to acknowledge structural processes and their impact on nursing, including racism and discrimination [64]. It is no wonder then that a Canadian study showed that being charged with discrimination did not appear to have hampered the careers of managers, while the careers of immigrant nurses of color who filed complaints were adversely affected [56].

Several limitations pertain to this study. First, the study did not examine the relationship between hours worked and income: although the women report high earnings, we cannot be definitive as to whether that is a function of qualifications or the long hours they are putting in. Expanding the sample size and collecting additional data on hours worked will help to clarify this issue. Further study will also have to look at how the women maintain work-life balance. A future study should go further in-depth to include more categories and variables such as marginalized identities and groups so as to highlight yet more nuances on the experiences of immigrant women in the United States. However, this study makes a valuable contribution to the immigrant narrative of African women. The resonating message in these experiences reflect strength, assertiveness, persistence and determination to overcome barriers to transcultural interaction in the workplace; valuing education and life-long learning; and managing experiences, perspectives and perceptions in a savvy manner [5]. The study has fleshed out the multiple pathways for immigrant women’s entry into the nursing profession that often involves adaptation of educational and professional choices in order to overcome entry barriers into the US labor market. It also highlights the importance of gender as a core analytical variable as it intersects with race, immigrant status and language in a continuously globalizing labor market. The paper has reaffirmed Waite and Nardi’s argument that racism is a key determinant in shaping the education of nursing students and influencing nursing practice [65]. As for diversity in the workplace, it raises the question: who should change? The tendency to argue that change should come from the diverse nurses only can result in discriminatory practices and racist assumptions that defend the status quo. Similarly, the inclination towards lip service about equity minimizes the problem of racial discrimination in employment [56]. Therefore, the successful integration of diverse nurses into the nursing profession requires change on all fronts so that difference is addressed in positive ways that recognize increasing diversity among nurses, a reality that all must adapt to [63].

## Figures and Tables

**Table 1:** Demographic Characteristics of Registered Nurses (RNs) Participants (N = 11).

**AGE:**	**FREQUENCY**
31–35	2
36–40	3
41–45	2
46–50	4
**EDUCATION (Years of Schooling):**	**FREQUENCY**
15–16	4
17–18	3
19+	4
**HIGHEST Educational Qualification for Current Job:**	**FREQUENCY**
Associate Degree in Nursing (ADN)	5
Bachelor of Science in Nursing Degree (BSN)	4
Master’s Degree	2
**MARITAL STATUS:**	**FREQUENCY**
Never Married	2
Married	7
Separated	1
Divorced	1
**NUMBER OFCHILDREN:**	**FREQUENCY**
0	2
1	3
2	5
4+	1
**TRAINED AS A NURSE OUTSIDE THE US?**	**FREQUENCY**
Yes	3
No	8
**LENGTH OF STAY IN THE US (YEARS):**	**FREQUENCY**
4–6 years	2
10+ years	9
**GROSS ANNUAL INCOME ($)**	**FREQUENCY**
40,000–59,999	1
60,000–79,999	7
80,000–99,999	3
